# EGFR-targeted photoimmunotherapy and its association with the immune microenvironment in locoregional recurrent head and neck squamous cell carcinoma

**DOI:** 10.3389/fimmu.2026.1814205

**Published:** 2026-04-10

**Authors:** Koichi Yoshizawa, Takahiro Tsujikawa, Peesit Leelasawatsuk, Mai Mohamed Bedeir, Yuna van der Aar, Tim de Martines, Saya Shibata, Hiroshi Ogi, Ryuhei Okada, Alisa Kimura, Hiroki Morimoto, Sumiyo Saburi, Shigeyuki Mukudai, Hikaru Nagao, Aya Miyagawa-Hayashino, Eiichi Konishi, Kyoko Itoh, Takahiro Asakage, Shigeru Hirano

**Affiliations:** 1 Department of Otolaryngology-Head and Neck Surgery, Kyoto Prefectural University of Medicine, Kyoto, Japan; 2 Department of Cell, Developmental and Cancer Biology, Oregon Health & Science University, Portland, OR, United States; 3 Department of Otolaryngology Head and Neck Surgery, Prince of Songkla University, Songkhla, Thailand; 4 SCREEN Holdings Co., Ltd., Kyoto, Japan; 5 Department of Pathology and Applied Neurobiology, Kyoto Prefectural University of Medicine, Kyoto, Japan; 6 Department of Head and Neck Surgery, Institute of Science Tokyo, Tokyo, Japan; 7 Department of Surgical Pathology, Kyoto Prefectural University of Medicine, Kyoto, Japan

**Keywords:** EGFR, head and neck squamous cell carcinoma, immune microenvironment, locoregional recurrence, photoimmunotherapy

## Abstract

**Background:**

Epidermal growth factor receptor (EGFR)-targeted photoimmunotherapy using cetuximab sarotalocan sodium (RM-1929) represents a novel therapeutic modality that induces selective tumor cell death and may influence the tumor immune microenvironment (TIME) in head and neck squamous cell carcinoma (HNSCC). However, its immunological consequences are not well defined.

**Methods:**

We retrospectively evaluated 25 patients with locoregional recurrent HNSCC who received RM-1929 photoimmunotherapy to evaluate treatment response, overall survival (OS), and safety. To explore TIME features, 14-marker multiplex immunohistochemistry was performed on pretreatment tumor samples from 14 patients, with 6 paired pre- and post-treatment samples.

**Results:**

Across 40 treatment cycles, the overall response rate was 77.5% (7 complete, 24 partial responses) with a median OS of 401 days. Treatment was generally well tolerated, with adverse events limited to expected local toxicities. Clinical responses were not explained by conventional clinicopathological factors or EGFR expression on tumor cells. In TIME analysis, responders exhibit significantly higher intratumoral CD39^+^CD8^+^ T cell density compared with non-responders. Longitudinal analyses revealed qualitative remodeling of the TIME after therapy, redistribution of CD8^+^ T cells and increasement of PD-L1^+^ immune cells.

**Conclusion:**

RM-1929 photoimmunotherapy demonstrated local efficacy with limited adverse events. Distinct TIME characteristics and potential immune remodeling, involving CD39^+^CD8^+^ T cell, were observed. These observations provide a rationale for further investigation of photoimmunotherapy as a strategy to integrate local tumor ablation with systemic immune modulation in recurrent HNSCC.

## Introduction

Locoregional recurrent head and neck squamous cell carcinoma (HNSCC) remains a persistent clinical challenge. Despite advances in chemotherapy and immunotherapy, patients with unresectable or recurrent disease have a median overall survival (OS) of 3–9 months (1–4). This poor prognosis underscores the urgent need for novel modalities that can achieve local tumor control while potentially engaging antitumor immunity. Recently, epidermal growth factor receptor (EGFR)-targeted photoimmunotherapy has emerged as a promising fifth treatment modality for HNSCC (5, 6). By coupling a tumor-specific antibody with a photoabsorbing dye, photoimmunotherapy delivers targeted near-infrared illumination to achieve selective tumor cell killing (5). Cetuximab sarotalocan sodium (RM-1929), a conjugate of cetuximab and IRDye700DX, targets the epidermal growth factor receptor (EGFR), which is overexpressed in 80–90% of HNSCC tumors (7). Following intravenous infusion, tumor sites are illuminated with near-infrared light. Recent study has reported that the photochemical reaction of IRDye700DX induces a rapid form of direct cellular damage termed photochemosis (8), whereby near-infrared light irradiation triggers aggregation of submembranous actin filaments. This aggregation disrupts the cytoskeletal support of the plasma membrane, leading to osmotic influx of water following the intracellular-extracellular osmotic gradient, resulting in acute cellular swelling, subsequent membrane rupture and localized necrosis.

Beyond direct cytotoxicity, preclinical studies have demonstrated that photoimmunotherapy promotes immunogenic cell death, characterized by calreticulin translocation to the cell surface and extracellular release of adenosine triphosphate, leading to dendritic cell maturation, CD8^+^ T cell priming, and durable antitumor memory (9, 10). In immunocompetent mouse models, a single photoimmunotherapy treatment achieved durable local tumor control, systemic antitumor immunity, and long-term immunological memory, highlighting its potential as an immune sensitizer (9). Furthermore, accumulating evidence from mouse models, as highlighted in recent studies, supports the notion that the concept of immunogenic cell death (ICD) entails the coordinated activation of innate and adaptive immunity, primarily mediated by the active or passive emission of damage-associated molecular patterns (10). These findings suggest that photoimmunotherapy may not only ablate tumors but also reshape the tumor–immune interface.

Despite these encouraging preclinical data, the immunologic consequences of photoimmunotherapy in the human tumor immune microenvironment remain insufficiently characterized. To address this gap, we retrospectively analyzed clinical outcomes and tumor specimens from patients with locoregional recurrent HNSCC treated with RM-1929 photoimmunotherapy at a single institution. Using multiplex immunohistochemistry and image cytometry (11), we profiled immune cell composition, functional phenotypes, and spatial architecture before and after treatment. This study was designed to link therapeutic response with TIME remodeling, thereby providing a rationale for future strategies that integrate photoimmunotherapy with systemic immunotherapies in recurrent HNSCC.

## Materials and methods

### Study population and sample collection

**Figure 1:**
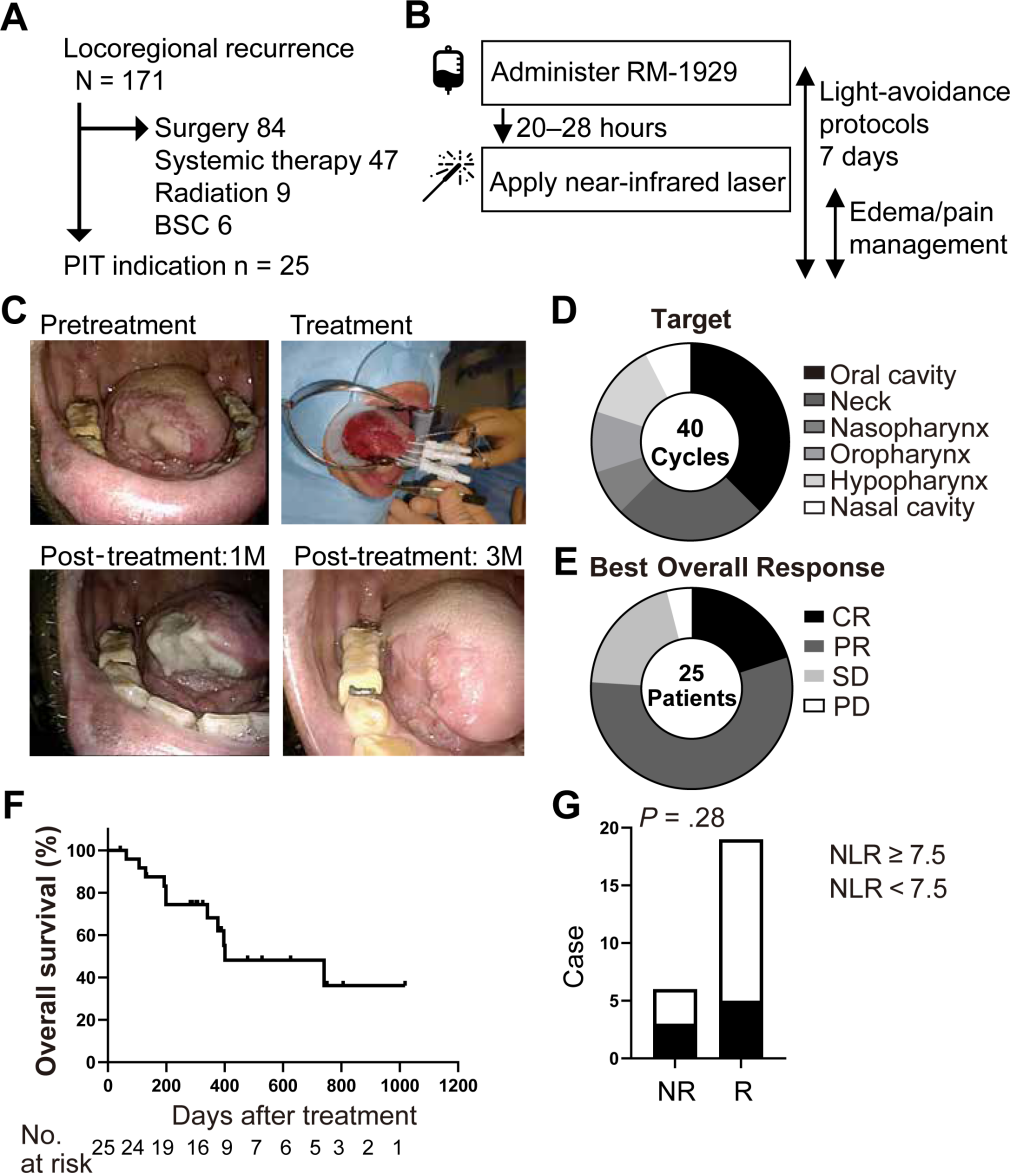
RM-1929 photoimmunotherapy demonstrates local treatment efficacy. **(A)** Flowchart summarizing the patient selection for photoimmunotherapy. Among 171 patients with locoregional recurrent HNSCC treated between December 2021 and May 2025, 25 patients were deemed to be eligible for undergoing RM-1929 photoimmunotherapy, based on clinical indications and tumor accessibility. **(B)** Schematic overview of the photoimmunotherapy protocol, including RM-1929 administration, light illumination, and perioperative clinical management timeline. **(C)** Representative photographs from a patient with tongue cancer: pre-treatment (upper left), day of treatment (upper right), 1-month post-treatment (lower left), and 3 months post-treatment (lower right). **(D)** A pie chart displaying the distribution of anatomical target sites among treatment cycles. **(E)** A pie chart showing the proportion of response outcomes per patient using modified RECIST criteria. CR, complete response; PR, partial response; SD, stable disease; PD, progressive disease. **(F)** Kaplan-Meier curves depicting overall survival (OS) of patients following photoimmunotherapy (median OS: 401 days). **(G)** Bar graph comparing the proportion of NR, non-responder and responder **(R)** cases stratified by a NLR, neutrophil-to-lymphocyte ratio cutoff value of 7.5; *P* = 0.28; OR, 2.8, by χ² test. Abbreviations: PIT, photoimmunotherapy; BSC, best supportive care.

This retrospective cohort study included all consecutive patients with locoregionally recurrent HNSCC who were treated and systematically followed at Kyoto Prefectural University of Medicine between December 1, 2021, and May 31, 2025. Of 171 screened patients, RM-1929 photoimmunotherapy were administered to 25 patients for unresectable locoregional recurrent disease, and received a total of 40 photoimmunotherapy cycles (**Figure 1A**). Patients were followed until death or the data cutoff date (January 31, 2026), whichever occurred first. Indications for RM-1929 photoimmunotherapy were considered based on the following criteria: histologically confirmed recurrent HNSCC deemed unsuitable for definitive surgery and radiation, and an Eastern Cooperative Oncology Group (ECOG) performance status of 0–2. Contraindications for RM-1929 photoimmunotherapy included tumors invading major blood vessels unless embolized, stented, or ligated; anatomical inaccessibility to light illumination; considerable hepatic or renal impairment; or a history of severe hypersensitivity reactions to cetuximab. The presence of distant metastatic disease was not considered an exclusion criterion for photoimmunotherapy.

For the exploratory tumor-immune profiling, formalin-fixed, paraffin-embedded (FFPE) tumor specimens were collected from fourteen patients by biopsy prior to their first photoimmunotherapy cycle. Specimen availability was restricted, as pretreatment samples had insufficient tissue due to cytology-based sampling (n = 6), or collected externally (n = 5). Six of these fourteen patients provided matched post-treatment specimens 3–5 months after photoimmunotherapy to evaluate post-treatment changes in the tumor-immune microenvironment.

### Treatment

RM-1929 photoimmunotherapy was administered according to a uniform institutional protocol. Each patient received a single intravenous infusion of RM-1929 at 640 mg/m², delivered 20–28 hours prior to light illumination. Target lesions were treated with a continuous-wave near-infrared laser light at 690 nm under general anesthesia using frontal or cylindrical diffusers, as appropriate (**Figure 1B**).

For superficial tumors (<1 cm thickness), the light exposure was fixed at 50 J/cm. For deeper lesions (≥1 cm), interstitial illumination was delivered using cylindrical diffusers at 100 J/cm per diffuser length. In these cases, 17-G needle catheters were inserted at 10–15 mm intervals throughout the tumor volume, guided by imaging modalities, such as ultrasonography, to ensure uniform coverage.

Prophylactic tracheostomy was performed in patients who underwent photoimmunotherapy for oropharyngeal or hypopharyngeal lesions to mitigate the risk of airway obstruction. Patients who did not undergo tracheostomy were monitored for oxygen saturation 24–48 hours postoperatively.

Pain control included intraoperative local anesthesia and postoperative intravenous patient-controlled analgesia for 12–48 hours, tailored to individual patient needs. To minimize phototoxic reactions, all patients were instructed to avoid exposing their skin and eyes to direct sunlight or bright indoor light for at least 4 weeks following infusion.

Baseline radiological evaluation was performed within 4 weeks of illumination using computed tomography or magnetic resonance imaging. Treatment response was prospectively defined and assessed at 4–12 weeks post-illumination by the treating physician according to the modified Response Evaluation Criteria in Solid Tumors version 1.1 (mRECIST 1.1), as defined in the phase 1/2 trial (5). The severity of adverse events was graded according to the Common Terminology Criteria for Adverse Events (CTCAE) version 5.0.

### Multiplex immunohistochemistry

Four µm FFPE sections were deparaffinized, rehydrated, and subjected to sequential multiplex immunohistochemistry as previously described (11). Slides were stained with a validated panel of 14 antibodies against immune cell lineages and tumor-specific markers (**Supplementary Tables 1**-**3**). Following chromogen development, the slides were scanned digitally at 20× objective magnification using a NanoZoomer S60 scanner (Hamamatsu Photonics, Shizuoka, Japan).

### Digital image processing and image cytometry

Following staining, image acquisition, and computational processing were performed as described previously. For image preprocessing, iteratively digitized images were co-registered using in-house software (SCREEN Holdings Co., Ltd.) by calculating the relative coordinates to a designated reference image. Regions of interest (ROIs) were extracted as non-compressed TIFF stacks. A heatmap of CD45^+^ cell density was used for selection of three rectangle ROIs within an intratumoral high CD45-density area (each ROI was approximately 6.25 mm^2^, and less than three if the analyzable cancerous area was covered more than 70%) (11). Co-registered images were split into single-marker channels, inverted, converted to grayscale, and pseudo-colored for visualization using Image-Scope Version 12.3.3.5048 (Leica Biosystems, Nussloch, Germany) and ImageJ. For quantitative image assessment, single-cell segmentation and quantification of staining intensity were performed using CellProfiler Version 2.2.0. All pixel intensity and shape-size measurements were exported in FCS Express 7 Image Cytometry–compatible format (v7.06.0015, DeNovo Software, Pasadena, CA, USA) for downstream image cytometry analysis of immune cell densities and spatial relationships.

### Spatial relationship analysis

Spatial proximity scores were calculated using previously published methods (12). Using fourteen FFPE samples with multiplex immunohistochemistry staining data, the spatial relationships between tumor cells and immune subsets were analyzed. X–Y coordinate data for pan-cytokeratin^+^ tumor cells, CD8^+^ T cells, and CD39^+^CD8^+^ T cells were extracted using FCS Express 7 Image Cytometry, where cell populations were gated and annotated based on marker expression, and their spatial positions were recorded for downstream analysis. Spatial associations were measured using the Spatstat package in RStudio 4.2.2 (Posit, Boston, MA, USA). Pair correlation functions were calculated over radial distances ranging from 5 µm to 30 µm from each cell center, and the area under the curve of pair correlation functions was calculated and normalized to g(r) = 1 to generate spatial proximity scores.

### Statistical analysis

Categorical variables were compared between outcome groups using the χ2 test. Odds ratios (ORs) and 95% confidence intervals (CIs) were calculated to estimate the strength of associations. Continuous variables were compared using the Kruskal-Wallis test. OS was estimated using the Kaplan-Meier method, with time-to-event defined from the date of first RM-1929 administration to death from any cause or censoring at the data cutoff date. Because of the limited sample size in the exploratory tumor-immune profiling, descriptive statistics were used to summarize outcomes. For pairwise comparison analyses, pairwise comparisons of median differences were assessed using the Wilcoxon matched-pairs signed rank test, with spearman method testing the effect of pairing statistically. Data analyses were performed using GraphPad Prism 10.5.0. (GraphPad Software, San Diego, CA, USA). Statistical significance was set at a 2-sided *P* < 0.05.

## Results

### Photoimmunotherapy demonstrated local treatment efficacy

We retrospectively analyzed 40 cycles in 25 patients with locoregionally recurrent HNSCC (19 males, 6 females; median age, 67 years) selected from a larger institutional cohort of 171 patients (**Figure 1A**). Baseline characteristics included a history of smoking in 18 patients (72%), daily alcohol consumption in 14 (56%), and 9 (36%) exhibited alcohol-induced facial flushing. Patients received an intravenous infusion of 640 mg/m² RM-1929 20–28 hours before near-infrared laser illumination under general anesthesia, with postoperative care focused on edema and pain control (**Figures 1B, C**). In the absence of carotid artery invasion, photoimmunotherapy was applied to multiple subsites of the head and neck, with the oral cavity identified as the most frequently targeted region (**Figure 1D**).

Responses were assessed using modified RECIST, applied exclusively to the target lesions. Patient-level responses were assessed by best overall response (BOR) resulted in 5 cases of complete response (CR), 14 cases of partial response (PR), 5 cases of stable disease (SD), and 1 case of progressive disease (PD), yielding an overall response rate (ORR) of 76.0% (**Figure 1E**). Cycle-level responses resulted in 7 CR, 24 PR, 7 SD, and 2 PD (**Supplementary Figure 1**). The median OS of the 25 patients was 401 days (**Figure 1F**). Treatment-related adverse events occurred in 32 of 40 treatment cycles (80%) and included grade 3 edema (n = 6), grade 3 pain (n = 1), and grade 3 delirium (n = 1) (**Supplementary Table 4**). Prophylactic tracheostomy was performed in eleven patients (44%). These findings confirmed that RM-1929 photoimmunotherapy could achieve high rates of local tumor control in unresectable HNSCC, while highlighting the cycles of SD and PD that warrant investigation into the determinants of response. These findings establish a clinical dataset of photoimmunotherapy outcomes, including both responders (CR/PR) and non-responders (SD/PD), that can serve as a reference for future translational studies.

### Photoimmunotherapy response was not associated with conventional clinicopathological factors

Next, we assessed whether the clinicopathological parameters correlated with the photoimmunotherapy response. Patients were classified as responders or non-responders based on their patient-level BOR across multiple treatment cycles, and compared according to age, sex, tumor subsite, histologic subtype, p16 status, smoking and alcohol history, alcohol-flush phenotype, baseline tumor diameter, prior treatments, neutrophil-to-lymphocyte ratio (NLR) (13), and incidence of treatment-related adverse events (**Table 1**). No clinicopathological parameters differed significantly between responders and non-responders. Although the median baseline tumor diameter was modestly larger in responders than in non-responders, this difference was not significant, indicating that the photoimmunotherapy efficacy was preserved regardless of the lesion size. Likewise, the frequency and severity of adverse events did not predict treatment outcomes.

**Table 1:** Clinicopathological parameters.

Variables	Non-responders (n = 6)	Responders (n = 19)	*P-*value
Sex			
Male	6 (100)	13 (68.4)	0.11
Female	0 (0)	6 (31.6)	
Age, median (standard deviation), years	75.5(15.5)	63(10.7)	0.20
Target lesion			
Oral cavity	2 (33.3)	7 (36.8)	0.19
Nasopharynx	0 (0)	1 (5.3)	
Oropharynx	0 (0)	3 (15.8)	
Hypopharynx	2 (33.3)	1 (5.3)	
Neck	2 (33.3)	5 (26.3)	
Skin	0 (0)	1 (5.3)	
Nasal cavity	0 (0)	1 (5.3)	
Primary subsite			
Oral cavity cancer	2 (33.3)	7 (36.8)	0.29
Sinonasal cancer	0 (0)	2 (10.5)	
Oropharyngeal cancer	1 (16.7)	6 (31.6)	
Hypopharyngeal cancer	2 (33.3)	4 (21.1)	
Laryngeal cancer	1 (16.7)	0 (0)	
Smoke, median (standard deviation), pack-years	8.5 (20.2)	25(26.7)	0.56
Heavy alcohol consumption			
Presence	2 (33.3)	12 (63.2)	0.20
Absence	4 (66.7)	7 (36.8)	
Alcohol-induced facial flushing			
Presence	4 (66.7)	5 (26.3)	0.07
Absence	2 (33.3)	14 (73.7)	
p16 status			0.23
Positive	1 (16.7)	2 (10.5)	
Negative	1 (16.7)	4 (21.1)	
Unknown	4 (66.7)	13 (68.4)	
Prior target, median (standard deviation), mm	22.3 (6.37)	23.1 (15.5)	0.61
Prior treatment			
None	1 (16.7)	5 (26.3)	0.46
Chemotherapy alone	1 (16.7)	2 (10.5)	
Immunotherapy alone	1 (16.7)	7 (36.8)	
Both chemotherapy and immunotherapy	3 (50)	5 (26.3)	
NLR, median (IQR1, 3)	5.7 (3.42, 8.73)	4.5(2.75, 7.56)	0.68
Adverse events			
None	2 (33.3)	3 (15.8)	0.22
Grade 1, 2	4 (66.7)	11 (57.9)	
Grade 3	0 (0)	5 (26.3)	

Statistical significance was determined using the χ2 and Kruskal-Wallis tests.

LN, lymph node; NLR, neutrophil-to-lymphocyte ratio; AE, adverse events; IQR, interquartile range.

Baseline NLR measured prior to each cycle tended to be higher in non-responder cycles than in responder cycles (median NLR: 5.7 vs 4.5), although the difference was statistically non-significant (*P* = 0.28; Odds Ratio, 2.8; 95% CI, 0.5–14.7) (**Figure 1G**, **Table 1**). No clear trend was observed in post-NLR measured after each cycle between non-responder and responder (median post-NLR: 4.6 vs 5.0) (**Supplementary Figure 2**). These findings suggested that conventional clinicopathological characteristics do not account for the heterogeneity in photoimmunotherapy responses, highlighting the importance of further investigating tumor characteristics as potential determinants of therapeutic efficacy.

### Investigation of tumor-immune microenvironment revealed baseline immune-inflamed profiles in photoimmunotherapy responders

**Figure 2:**
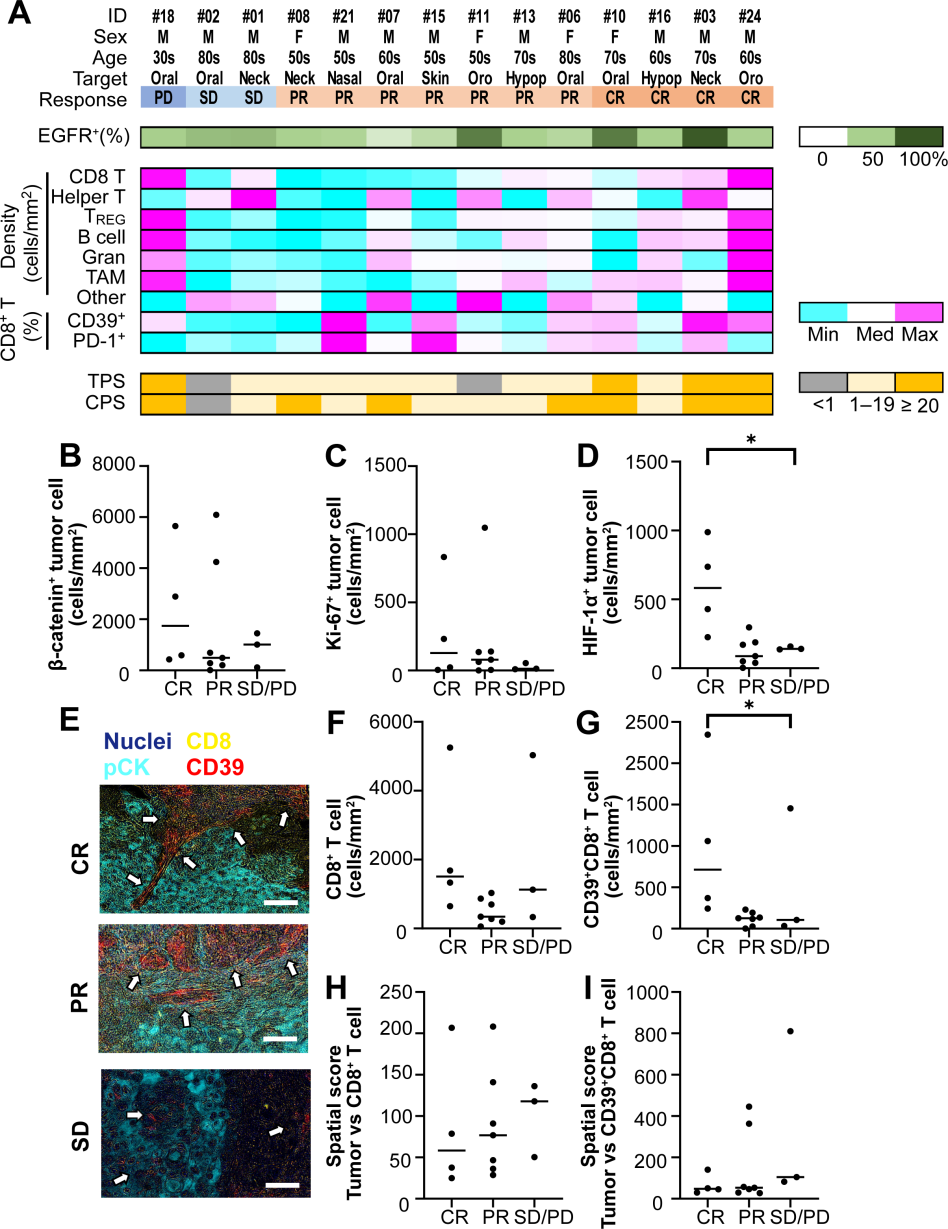
Baseline tumor-immune microenvironment and its association with photoimmunotherapy response. **(A)** Heatmap summarizing immune complexity profiles from fourteen pre-treatment tumor samples. EGFR, Epidermal growth factor receptor ^+^ percentages of pan-cytokeratin (pCK)^+^ tumor cells, immune cell densities, CD39^+^ and PD-1^+^ percentages of CD8^+^ T cells, and TPS, total proportion score and CPS, combined positive score of PD-L1 expression are presented, along with the relevant scales. **(B–D)** Quantitative comparison of unfavorable prognostic markers across clinical response groups. Scatter plots show densities of **(B)** β-catenin (*P* = 0.74), **(C)** Ki-67 (*P* = 0.61), and **(D)** HIF-1α (*P* = 0.013) in complete response (CR), partial response (PR), and stable or progressive disease (SD/PD) cases. Bars indicate group medians. **(E)** Representative multiplex images displaying cellular composition in CR, PR, and SD cases. White arrows indicate CD39^+^CD8^+^ T cells. Scale bar = 100 μm.(**F–G**) Scatter plots comparing densities of CD8^+^ T cells **(F)** and CD39^+^CD8^+^ T cells **(G)** across clinical response groups. CD39^+^CD8^+^ T cell density was significantly higher in CR cases (*P* = 0.032); CD8^+^ T cell density does not differ (*P* = 0.061). (**H–I**) Spatial proximity scores between tumor cells and immune subsets. Scatter plots show average distances for **(H)** CD8^+^ T cells (*P* = 0.76) and **(I)** CD39^+^CD8^+^ T cells (*P* = 0.25) across clinical response groups. Higher spatial scores indicate closer proximity to tumor cells. Statistical significance was assessed using the Kruskal-Wallis test. Pairwise comparisons of median differences were assessed using the Hodges-Lehmann method, with corresponding 95% CIs. *P < 0.05.

Given that an elevated NLR is a surrogate for systemic inflammatory burden and immune suppression (13), we hypothesized that the local tumor-immune context may underlie clinical outcomes. Pre-treatment biopsies from fourteen patients (four CR, seven PR, two SD, and one PD) were subjected to 14-marker multiplex immunohistochemistry and quantitative image cytometry (**Supplementary Figures 3A–N**) (11). Responders exhibited trends toward higher EGFR expression, CD39^+^ CD8^+^ T cells, programmed cell death ligand-1 (PD-L1) expression (14, 15), and combined positive scores (CPS) (14, 15) than non-responders (**Figure 2A**). However, EGFR expression (7, 16) was uniformly high across all cases (median 71.5%, 61.9%, and 77.9% for CR, PR, and SD/PD, respectively), indicating that EGFR expression alone could not predict the efficacy of photoimmunotherapy (17) (**Figure 2A**). We then assessed markers of tumor aggressiveness, including β-catenin (18) and Ki-67 (19), and found no significant differences in their expression between response groups (**Figures 2B, C**). This finding suggested that tumor intrinsic markers are unlikely to account for differential photoimmunotherapy outcomes. However, hypoxia-inducible factor-1α (HIF-1α) expression, which is typically related to hypoxic environment (20), was significantly higher in CR cases than in PR and SD/PD cases (**Figure 2D**), suggesting that hypoxic microenvironmental states might be linked to enhanced photoimmunotherapy efficacy.

Focusing on intratumoral T cell populations, we quantified the CD8^+^ and CD39^+^CD8^+^ T cell densities between response groups (**Figure 2E**). CR cases exhibited a tendency of higher total CD8^+^ T cell density than PR or SD/PD cases (**Figure 2F**). Notably, CR cases demonstrated significantly higher CD39^+^CD8^+^ T cell density than PR or SD/PD cases (CR vs PR, 588.3; CR vs SD/PD, 609.9; PR vs SD/PD, 21.6) (**Figure 2G**). Spatial proximity analysis, performed as reported previously (12), revealed that complete responders tended to exhibit lower spatial scores than PR or SD/PD cases, indicating greater separation between CD39^+^CD8^+^ T cells and tumor cells (**Figures 2H, I**). Given that CD39^+^CD8^+^ T cells frequently represent chronically stimulated, antigen-experienced T cell subsets (21), their preferential localization to peritumoral regions may reflect focused recruitment or retention of effector cells poised to recognize tumor antigens. Collectively, we identified CD39^+^CD8^+^ T cell abundance and spatial distribution as candidate immune biomarkers for predicting response to RM-1929 photoimmunotherapy.

### Longitudinal changes in the tumor-immune microenvironment and immunogenic cell death marker following photoimmunotherapy

**Figure 3:**
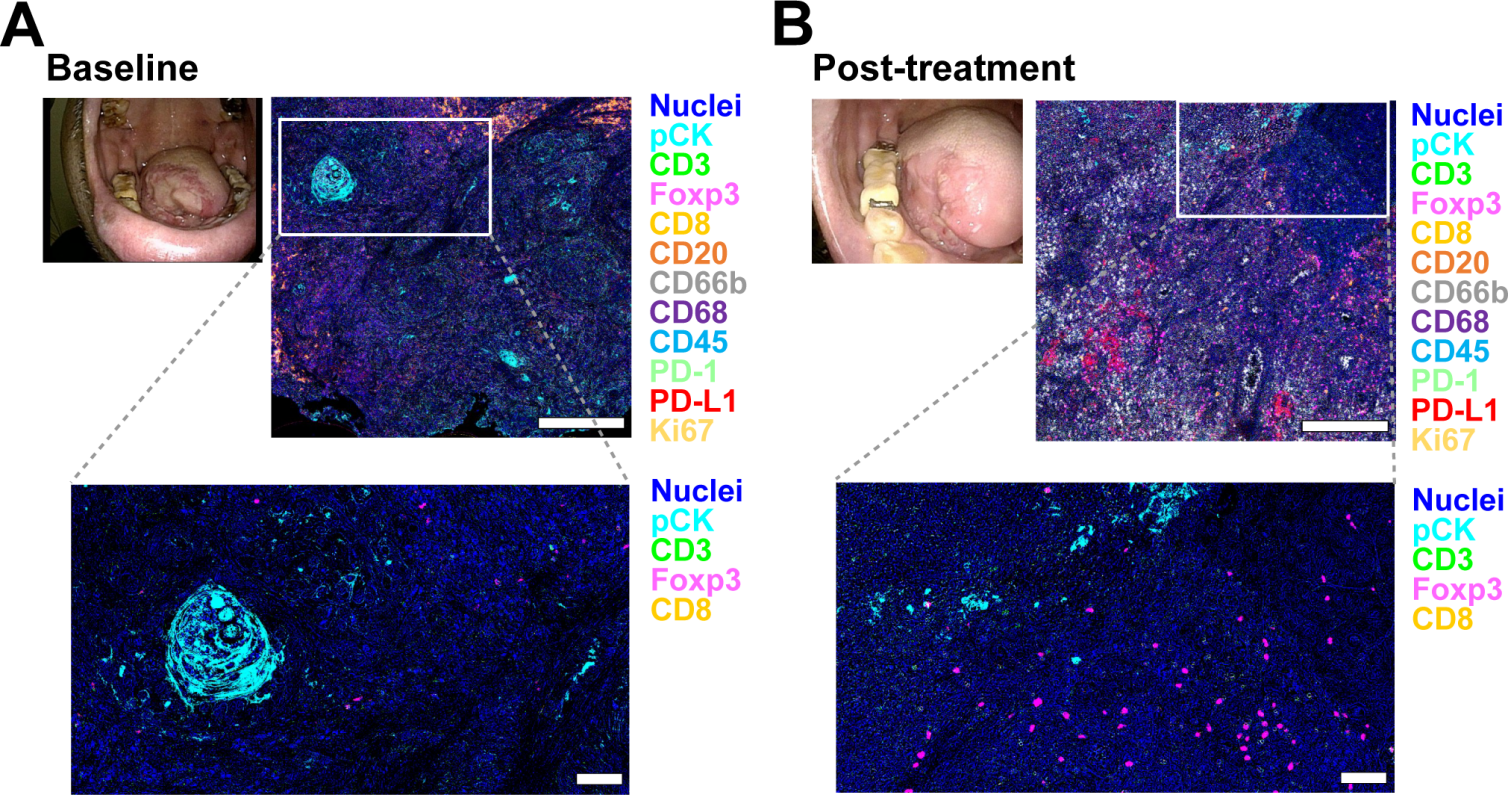
Longitudinal changes in the tumor-immune microenvironment between baseline and post-photoimmunotherapy. **(A, B)** Fourteen-marker multiplex immunohistochemistry images from pre-treatment **(A)** and post-treatment biopsy **(B)** following RM-1929 photoimmunotherapy. The boxed regions (upper images) indicate the areas shown at higher magnification in the lower images. Scale bar: upper panel = 500 µm; lower panels = 100 µm.

**Figure 4:**
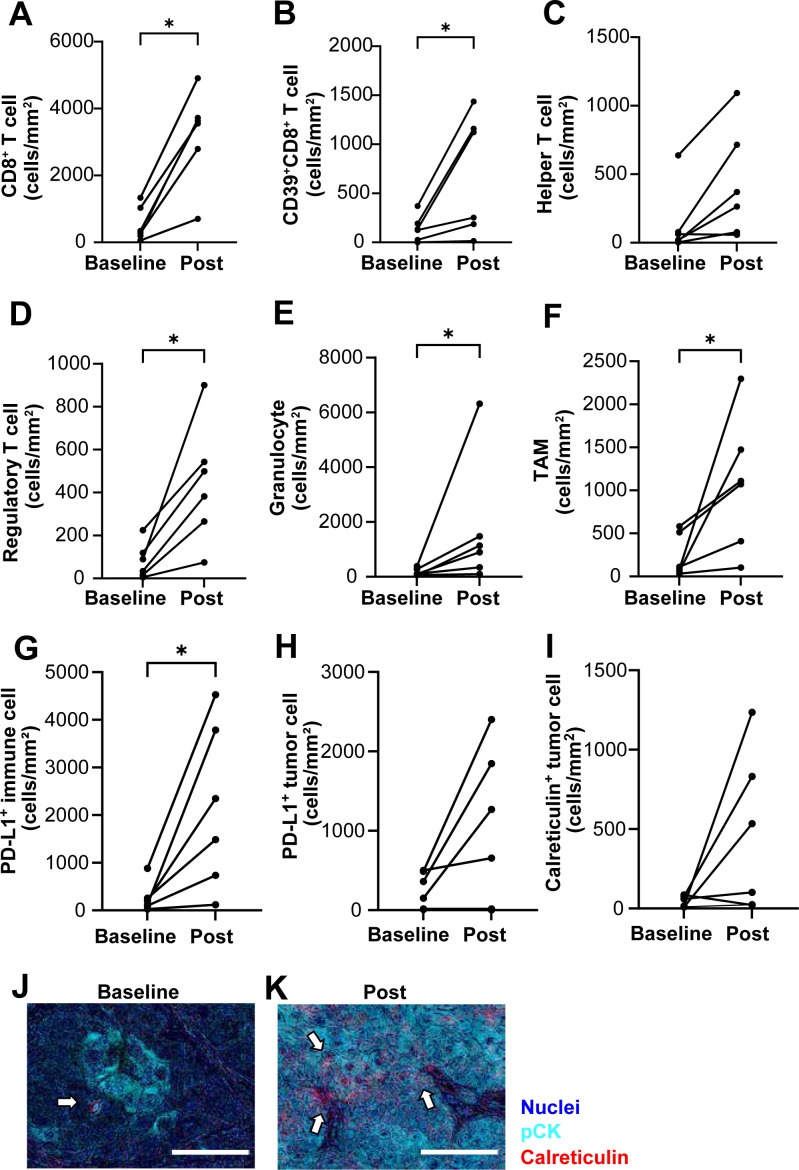
Immune cell density, PD-L1 expression, and calreticulin upregulation following RM-1929 photoimmunotherapy. **(A–H)** Line graphs showing the density of immune cell subsets, including CD8^+^ T cells (two-tailed exact *P* = 0.031, rs = 0.6 one-tailed *P* = 0.12) **(A)**, CD39^+^CD8^+^ T cells (two-tailed exact *P* = 0.031, rs = 1.0 one-tailed *P* = 0.0014) **(B)**, helper T cells (two-tailed exact *P* = 0.063, rs = 0.6 one-tailed *P* = 0.12) **(C)**, regulatory T cells (Tregs) (two-tailed exact *P* = 0.031, rs = 0.83 one-tailed *P* = 0.029) **(D)**, granulocytes (two-tailed exact *P* = 0.031, rs = 0.6 one-tailed *P* = 0.12) **(E)** and TAMs, tumor-associated macrophages (two-tailed exact *P* = 0.031, rs = 0.14 one-tailed *P* = 0.40) **(F)**. **(G, H)** Line graphs showing the density of PD-L1 expressed cells in immune cells (two-tailed exact *P* = 0.031, rs = 0.77 one-tailed *P* = 0.051) **(G)** and in tumor cells (two-tailed exact *P* = 0.031, rs = 0.66 one-tailed *P* = 0.088) **(H)** based on multiplex immunohistochemistry. **(I)** Line graphs showing the density of calreticulin expression before and after photoimmunotherapy (two-tailed exact *P* = 0.16, rs = -0.26 one-tailed *P* = 0.33). **(J–K)** Representative images of calreticulin expression in pre-treatment **(J)** and post-treatment **(K)** tumor samples. White arrows indicate calreticulin^+^ cells. Scale bar = 100 µm. Pairwise comparisons of median differences were assessed using the Wilcoxon matched-pairs signed rank test, with spearman method testing the effect of pairing statistically. *P < 0.05.

As patients who achieved CR displayed a more pronounced inflammatory profile than those who had PR, SD or PD (**Figure 2A**), we performed a paired, longitudinal analysis of the six responders. To evaluate dynamic changes in the TIME, we performed paired pre- and post-photoimmunotherapy specimen analyses in six patients (one CR and five PR cases) (**Figures 3A, B**). At baseline, six cases exhibited low densities of total immune cells, characterized by scarce T cell infiltrates (**Figures 4A–F**). Following photoimmunotherapy, we observed increased densities in CD8^+^ T cells, CD39^+^CD8^+^ T cells, regulatory T cells (Tregs), granulocyte and tumor-associated macrophages (**Figures 4A, B, D–F**), along with an elevation in PD-L1^+^ immune cell density (**Figures 4G, H**), potentially reflecting a shift toward immunological engagement with compensatory checkpoint expression. A Wilcoxon matched-pairs signed-rank test demonstrated a clear trend between the paired groups of CD39^+^CD8^+^ T cell density (two-tailed, exact *P* = 0.0312). The median difference was 563.0 (96.88% confidence interval, 13.36-1066). The effectiveness of this pairing was confirmed by Spearman’s pair correlation analysis (rs = 1.000, one tailed *P* = 0.0014) (**Figure 4B**). These findings were consistent with the outcome previously reported in Phase Ib/II Study (22). Importantly, calreticulin (23) expression, which is related to ICD (23–25), tended to increase after treatment (median: 38.3 vs 318.5, 283.5) (**Figures 4I–K**). These longitudinal observations provide a dataset linking clinical outcomes with immune microenvironmental remodeling, offering a resource for investigating mechanisms of response and resistance to RM-1929 photoimmunotherapy (23–25).

Together, this resource combines a systematically followed clinical cohort, standardized treatment protocols, and high-dimensional immune profiling of tumor specimens from patients treated with RM-1929 photoimmunotherapy. These datasets not only provide insights into the local and systemic effects of photoimmunotherapy but also establish a platform for future research. Specifically, the availability of paired pre- and post- treatment samples, single-cell spatial immune data, and linked clinical outcomes offers opportunities to refine biomarkers of therapeutic response, to develop computational models of tumor-immune interactions, and to guide the rational design of combination strategies integrating photoimmunotherapy with checkpoint blockade or other immunotherapies. By sharing this resource, we aim to facilitate collaborative efforts toward advancing precision oncology in HNSCC.

## Discussion

In this study, we retrospectively evaluated 40 treatment cycles of RM-1929 photoimmunotherapy administered to 25 patients with locoregional recurrent HNSCC, and complemented clinical outcomes with multiplex immunohistochemistry-based profiling in a subset of fourteen cases. Together, these data provide a clinical and immunopathological resource for the community to better understand the determinants of photoimmunotherapy response. At the clinical level, RM-1929 photoimmunotherapy achieved a local response rate (ORR 77.5%) and a median OS of 401 days, consistent with the findings of prior studies, underscoring its promise for patients with unresectable disease (1–4). Although no statistically significant difference was observed, NLR tended to be higher in non-responders, suggesting that NLR might represent a potential surrogate marker of systemic immune suppression and warrants further evaluation in larger cohorts.

The favorable efficacy of photoimmunotherapy in our cohort is comparable to the historical outcomes for multimodal therapies in locoregional recurrent HNSCC (**Figures 1E, F**), where the median OS typically ranges from 9 to 12 months and ORRs frequently remain below 50% (2–4). Importantly, adverse events were manageable and predominantly transient, supporting the feasibility of this modality in patients with unresectable disease (**Table 1**). While these findings reaffirm the therapeutic potential of RM-1929 photoimmunotherapy, they also underscore the need to define biological correlates that distinguish responders from non-responders. Although pack−years did not significantly differ between responders and non−responders (**Table 1**), smoking remains an important biological factor to consider when interpreting treatment response in HNSCC. Beyond its well−established epidemiologic role in carcinogenesis, recent mechanistic studies have shown that nicotine exposure can directly promote tumor progression and recurrence, thereby shaping the tumor microenvironment in ways that may influence therapeutic resistance (26). Conversely, smoking−associated mutagenesis is known to increase tumor mutational burden, which can enhance neoantigen availability and potentially augment responsiveness to immunotherapy (27, 28). These opposing biological effects may help explain why no clear directional association between smoking history and treatment outcome was observed in our cohort.

Consistent EGFR expression across the evaluated cases supported the reliance of RM-1929 on cetuximab-mediated targeting and supported repeated administration in EGFR-positive tumors (**Figure 2A**). Conversely, levels of Ki-67 and β-catenin expression were not associated with clinical outcomes, suggesting that conventional proliferative or hypoxia-related biomarkers may have limited predictive value for photoimmunotherapy (**Figures 2B, C**). Hypoxia is generally associated with poor prognosis and reduced efficacy of conventional chemotherapy and radiotherapy (29). However, our study unexpectedly suggested enhanced treatment efficacy under hypoxic conditions. Previous studies have suggested that increased susceptibility of the hydrophilic ligand of IR700 under hypoxic conditions promote dissociation of its side chain of IR700 through NIR irradiation (30, 31) (**Figure 2D**). After dissociation, the chemical structure of IR700 changes, and the radical anion is produced preferably in hypoxic conditions (32). In this context, pre-treatment HIF-1α expression in complete responders might reflect a hypoxic tumor microenvironment that enhances ligand dissociation, thereby potentially contributing to improved therapeutic efficacy. Further studies using animal models will be required to validate this hypothesis.

RM-1929 photoimmunotherapy is a novel treatment for locoregional recurrent HNSCC that encompasses two mechanisms: direct cellular injury caused by photochemosis (8) and subsequent ICD. Although the number of clinical cases remains limited, this study represents the first report to investigate TIME using actual human specimens from these rare cases, and the findings were consistent with previously reported observations. Our tumor-immune analyses highlighted the pivotal role of local CD8^+^ T cell subsets in modulating the treatment response because CD8^+^ T cells are known to play a crucial role in antitumor immunity by recognizing antigens presented from destroyed cancer cells induced by photochemosis (**Figure 2A**). Complete responders displayed significantly higher densities of CD39^+^ CD8^+^ T cells and tendency of increased mean distances between these cells and tumor cells compared with patients with PR and SD/PD (**Figures 2G, I**). This spatial pattern suggests that chronically activated antigen-experienced T cells preferentially localize to peritumoral niches (33, 34), poised for rapid engagement upon antigen release. Notably, preclinical studies have demonstrated that CD39^+^CD8^+^ T cells are functionally linked to tumor-specific immune recognition and may serve as key mediators of durable antitumor responses in settings of immunogenic cell death, as induced by photoimmunotherapy-like modalities (35). These results nominate CD39^+^CD8^+^ T cell topography as a candidate biomarker of sensitivity to photoimmunotherapy, aligning with preclinical data linking this subset to antigen-specific immune recognition and durable tumor control.

Longitudinal biopsies further revealed the potential of photoimmunotherapy for reprogramming the TIME. Post-treatment specimens exhibited increased infiltration of immune cells including CD8^+^ T cells and elevated PD-L1^+^ immune cell density, accompanied by marked upregulation of calreticulin, a well-known ICD marker (23) (**Figures 4C, D**). ICD is generally regarded as an acute event occurring within 24 hours after treatment (36). However, Kroemer et al. describe that ICD−related signals can also arise during later phases of tumor evolution when ongoing tumor cell turnover, immune−mediated cytotoxicity, or secondary necrosis persists within the tumor microenvironment (37). In this study, post-treatment specimens were obtained 3–5 months after therapy, and the observed increase in calreticulin expression may therefore reflect sustained immune activity or prolonged tissue remodeling rather than acute photochemical effects alone. Previous report in phase Ib/II study showed temporal changes in CD8^+^ T cell populations following photoimmunotherapy (22), supporting the notion that immune dynamics can evolve over extended periods. These dynamic changes support a model in which photoimmunotherapy not only delivers targeted cytotoxicity, but also promotes immunogenic cell death, potentially enhancing dendritic cell maturation and antigen presentation (9, 23–25). While the real−world timing of biopsies limits precise mechanistic interpretation, these findings suggest that photoimmunotherapy may influence the tumor immune milieu beyond the immediate post−treatment window, warranting further investigation with predefined sampling time points. This immunogenic remodeling may synergize with checkpoint blockade to prolong systemic antitumor immunity (38).

The principal limitation of this dataset is the retrospective and single-center design, with a small number of evaluable tissue profiling. Moreover, the post-treatment specimens were collected only after prolonged interval of 3–5 months following photoimmunotherapy, owing to the challenges in securing post-treatment specimens. However, anti-tumor host immunity might be enhanced after RM1929-photoimmunotherapy primarily by re-education and subsequent expansion of CD8^+^ T cells in TIME, after neoantigens are released (39). Post-treatment specimen in this study demonstrated newly enhanced anti-tumor host immunity reflecting re-education of immune cells. Nevertheless, by integrating clinical outcomes with high-dimensional tissue profiling, this work establishes a foundation for future biomarker validation, mechanistic studies of immunogenic cell death, and rational design of combinatorial strategies. Prospective studies with larger and more diverse populations are needed to validate our proposed immune biomarkers and clarify the interplay between photoimmunotherapy-induced immunogenic cell death and adaptive antitumor responses.

In summary, we provide a combined clinical and tissue-based dataset of RM-1929 photoimmunotherapy in recurrent HNSCC. Differential TIME characteristics and potential immune remodeling, involving CD39^+^CD8^+^ T cell, were observed. These observations provide a rationale for further investigation of photoimmunotherapy as a strategy to integrate local tumor ablation with systemic immune modulation in recurrent HNSCC. We aim to support the research community in advancing biomarker discovery, refining immunotherapeutic approaches, and ultimately improving the treatment of this challenging disease. 

## Data Availability

The original contributions presented in the study are included in the article/**Supplementary Material**. Further inquiries can be directed to the corresponding author.
