# Supplementary material for: EGFR-targeted photoimmunotherapy and its association with the immune microenvironment in locoregional recurrent head and neck squamous cell carcinoma
Source: Front Immunol. 2026 Apr 10;17:1814205. doi: 10.3389/fimmu.2026.1814205 (PMC13106176; doi:10.3389/fimmu.2026.1814205)
Supplement: Supplementary file 1 [file DataSheet1.pdf]

Supplementary Material

1 Supplementary Data

Supplementary Figure 1

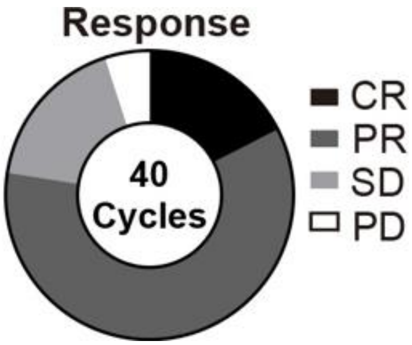

Supplementary Figure 1. Response outcomes of 40 cycles

A pie chart showing the proportion of response outcomes per treatment cycle using modified RECIST criteria. CR, complete response; PR, partial response; SD, stable disease; PD, progressive disease.

Supplementary Figure 2

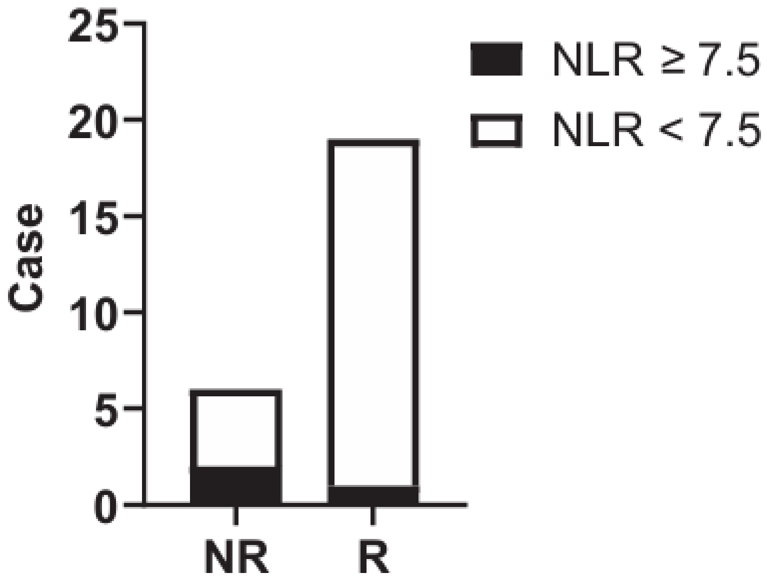

Supplementary Figure 2. Relevance of the post-treatment NLR and clinical responses

Bar graph comparing the proportion of non-responder (NR) and responder (R) cases stratified by a post-neutrophil-to-lymphocyte ratio (post-NLR) cutoff value of 7.5

Supplementary Figure 3

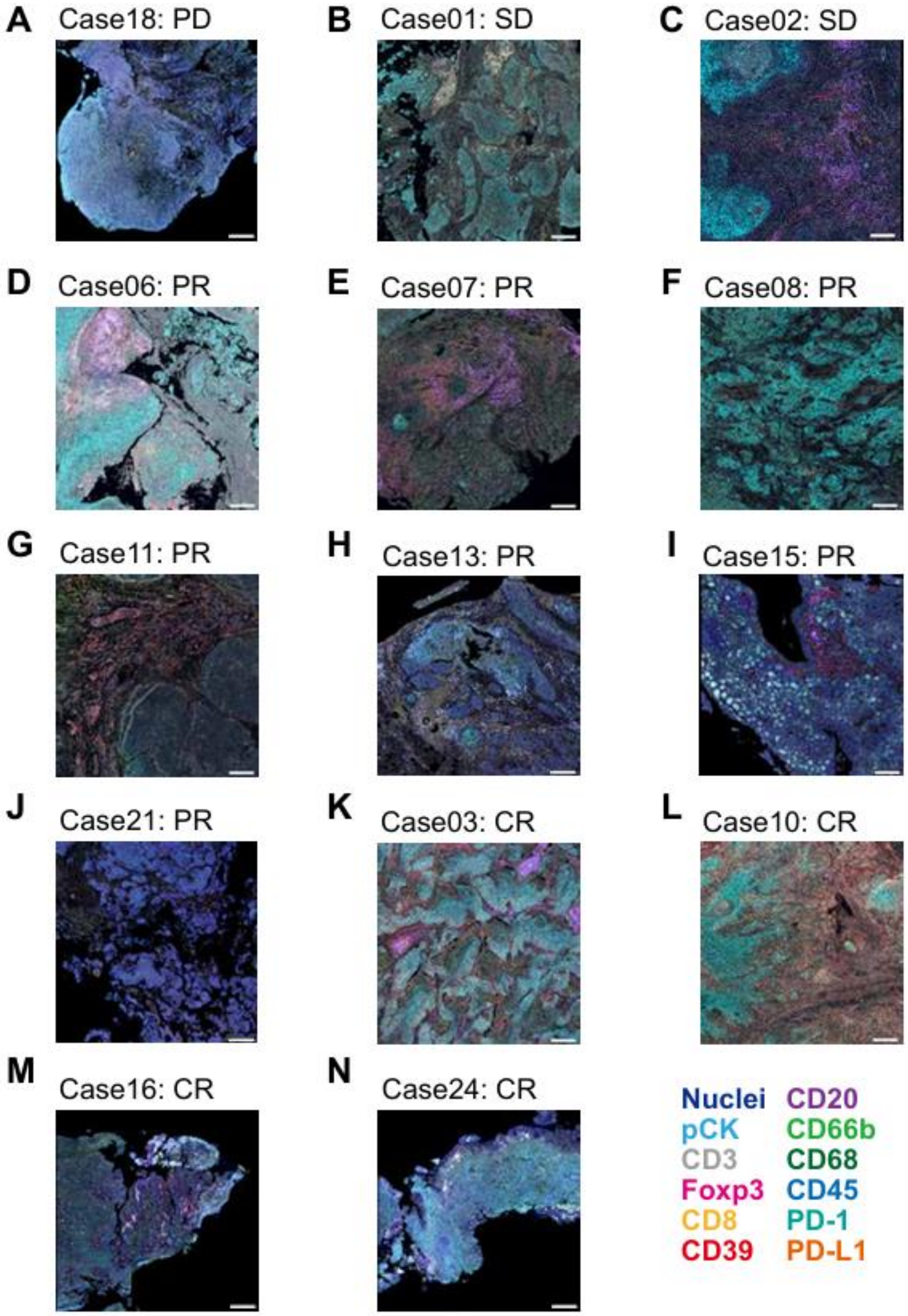

Supplementary Figure 3. All stained single marker data of 14 patients

(A-N) Multiplex immunohistochemistry images from pre-treatment. Scale bar: 500 µm. Abbreviations: PD, progressive disease; SD, stable disease; PR, partial response; CR, complete response; pCK, pan-cytokeratin.

Supplementary Table 1. A list of antibodies and conditions used for immune cell panel

	Cycle1	Cycle2	Cycle3	Cycle4	Cycle5	Cycle6	Cycle7
Primary Ab	Hematoxylin	PD1	CD8	CD3	CD68	LAG3	CD45
Clone/Product#		NAT105	C8/144B	SP7	PG-M1	EPR4392	H130
Vender	Dako	Abcam	Thermo science	Thermo science	Abcam	Abcam	Thermo science
Conc		1/50	1/100	1/50	1/50	1/1000	1/100
Reaction	2min	RT, 30min	RT, 30min	RT, 30min	RT, 30min	RT, 30min	RT, 30min
Secondary Ab		Anti-mouse	Anti-mouse	Anti-rabbit	Anti-mouse	Anti-rabbit	Anti-mouse
Reaction		RT, 30min	RT, 30min	RT, 30min	RT, 30min	RT, 30min	RT, 30min
AEC			20min		20min	20min	20min
AMEC		5min		5min			

	Cycle8	Cycle9	Cycle10	Cycle11	Cycle12	Cycle13	Cycle14
Primary Ab	PD-L1	CD39	Foxp3	CD20	CD66b	Ki-67	M aPan-CK
Clone/Product#	ElL3N	EPR20627	236A/E7	L26	G10F5	SP6	AE1/AE3
Vender	Cell signaling	Abcam	eBioscience	Abcam	eBioscience	Cell marque	Abcam
Conc	1/100	1/1000	1/40	1/200	1/400	1/2000	1/2000
Reaction	4℃, overnight	RT, 30min	RT, 30min	RT, 30min	RT, 30min	RT, 30min	RT, 30min
Secondary Ab	Anti-rabbit	Anti-rabbit	Anti-mouse	Anti-mouse	Anti-mouse	Anti-rabbit	Anti-mouse
Reaction	RT, 30min	RT, 30min	RT, 30min	RT, 30min	RT, 30min	RT, 30min	RT, 30min
AEC		20min	20min	20min	20min	20min	20min
AMEC	5min						

Abbreviations: Pan-CK, pan-cytokeratin; RT, room temperature.

Supplementary Table 2. A list of antibodies and conditions used for tumor characteristics panel

	Cycle1	Cycle2	Cycle3	Cycle4	Cycle5	Cycle6	Cycle7	Cycle8	Cycle9	Cycle10	Cycle11
Primary Ab	Hem	HIF1A	Bcatenin	CD3	α-SMA	CD68	Ki-67	pCK	EGFR	Calreticulin	R aCCP3
Clone/Product#		ab114977	ab16051	SP7	ab5694	PG-M1	SP6	AE1/AE3	SP84	EPR3924	ASP175
Vender		Abcam	Abcam	Thermo science	Abcam	Abcam	Cell marque	Abcam	Abcam	Abcam	Cell Signaling
Conc		1/75	1/300	1/100	1/200	1/50	1/2000	1/1000	1/50	1/5000	1/400
Reaction		RT, 30min	RT, 30min	RT, 30min	RT, 30min	RT, 30min	RT, 30min	RT, 30min	RT, 30min	RT, 30min	4°C, overnight
Secondary Ab		Anti-rabbit	Anti-rabbit	Anti-rabbit	Anti-rabbit	Anti-mouse	Anti-rabbit	Anti-mouse	Anti-rabbit	Anti-rabbit	Anti-rabbit
Reaction		RT, 30min	RT, 30min	RT, 30min	RT, 30min	RT, 30min	RT, 30min	RT, 30min	RT, 30min	RT, 30min	RT, 30min
AEC		20min	20min	20min	20min	20min	20min	20min	20min	20min	20min

Abbreviations: Pan-CK, pan-cytokeratin; RT, room temperature.

Supplementary Table 3. A list of antibodies and conditions used for additional tumor characteristics panel

	Cycle1	Cycle2	Cycle3	Cycle4
Primary Ab	Hem	HSP70	M aNKp46	pCK
Clone/Product#		4872	195314	AE1/AE3
Vender		Cell signaling	R & D	Abcam
Conc		1/600	1/200	1/1000
Reaction		4°C, overnight	RT, 30min	RT, 30min
Secondary Ab		Anti-rabbit	Anti-mouse	Anti-mouse
Reaction		RT, 30min	RT, 30min	RT, 30min
AEC		20min	20min	20min

Abbreviations: Pan-CK, pan-cytokeratin; RT, room temperature.

Supplementary Table 4. Treatment-related adverse events in 40 treatment cycles

CTCAE v5.0	All grades, n (%)	Grade ≥ 3	Grade ≥ 4
Any TEAE	32 (80.0)	8 (20.0)	–
Tumor pain	25 (62.5)	1 (2.5)	–
Localized edema	14 (35.0)	6 (15.0)	–
Facial edema	5 (12.5)	–	–
Tumor hemorrhage	2 (5.0)	–	–
Delirium	2 (5.0)	1 (2.5)	–
Accessory nerve disorder	1 (2.5)	–	–

Abbreviations: CTCAE, common terminology criteria for adverse events; TEAE, treatment-emergent adverse events.
